# Supplementary material for: Hot spot prediction in protein-protein interactions by an ensemble system
Source: BMC Syst Biol. 2018 Dec 31;12(Suppl 9):132. doi: 10.1186/s12918-018-0665-8 (PMC6311905; doi:10.1186/s12918-018-0665-8)
Supplement: Supplementary file 2 — All relevant MATLAB code. A simple Matlab implement of our predictor. (ZIP 19,742 kb) [file 12918_2018_665_MOESM2_ESM.zip › Additional file 2/Read ASA/ASA website/SKEMPI/1DANH.htm]

 NetSurfP - Protein Surface Accessibility and Secondary Structure Predictions

  

|  |  |  |
| --- | --- | --- |
|  |  | NetSurfP - Protein Surface Accessibility and Secondary Structure Predictions  Technical University of Denmark |

  


---

```
# For publication of results, please cite:
# A generic method for assignment of reliability scores applied to solvent accessibility predictions.
# Bent Petersen, Thomas Nordahl Petersen, Pernille Andersen, Morten Nielsen and Claus Lundegaard
# BMC Structural Biology 2009, 9:51 doi:10.1186/1472-6807-9-51
#
# Column 1: Class assignment - B for buried or E for Exposed - Threshold: 25% exposure, but not based on RSA
# Column 2: Amino acid
# Column 3: Sequence name
# Column 4: Amino acid number
# Column 5: Relative Surface Accessibility - RSA
# Column 6: Absolute Surface Accessibility
# Column 7: Z-fit score for RSA prediction
# Column 8: Probability for Alpha-Helix
# Column 9: Probability for Beta-strand
# Column 10: Probability for Coil
E I  1DANH                  1    0.360  66.674  -2.195   0.005   0.045   0.951
B V  1DANH                  2    0.168  25.883  -2.296   0.004   0.514   0.481
E G  1DANH                  3    0.415  32.660  -0.895   0.005   0.336   0.660
B G  1DANH                  4    0.270  21.241  -1.569   0.020   0.205   0.775
E K  1DANH                  5    0.552 113.546   1.165   0.021   0.279   0.699
E V  1DANH                  6    0.593  91.221   1.300   0.020   0.205   0.775
B C  1DANH                  7    0.058   8.101  -0.226   0.018   0.088   0.893
E P  1DANH                  8    0.564  79.975   1.403   0.018   0.047   0.935
E K  1DANH                  9    0.517 106.367   0.214   0.018   0.047   0.935
E G  1DANH                 10    0.536  42.167  -0.336   0.018   0.019   0.964
E E  1DANH                 11    0.511  89.254   1.026   0.018   0.019   0.964
B C  1DANH                 12    0.167  23.475   1.071   0.018   0.088   0.893
B P  1DANH                 13    0.105  14.857   0.892   0.021   0.279   0.699
B W  1DANH                 14    0.062  14.815   0.445   0.021   0.756   0.223
B Q  1DANH                 15    0.063  11.288   0.732   0.011   0.918   0.071
B V  1DANH                 16    0.022   3.351   0.858   0.011   0.918   0.071
B L  1DANH                 17    0.036   6.665  -0.009   0.011   0.918   0.071
B L  1DANH                 18    0.023   4.193   1.059   0.011   0.918   0.071
B L  1DANH                 19    0.144  26.293   0.619   0.011   0.918   0.071
B V  1DANH                 20    0.215  33.092   0.450   0.023   0.655   0.322
E N  1DANH                 21    0.529  77.387  -0.319   0.018   0.088   0.893
E G  1DANH                 22    0.689  54.232  -2.004   0.005   0.015   0.979
E A  1DANH                 23    0.453  49.899   0.295   0.004   0.197   0.799
B Q  1DANH                 24    0.120  21.378  -1.115   0.004   0.420   0.576
B L  1DANH                 25    0.127  23.345   0.551   0.003   0.718   0.279
B C  1DANH                 26    0.016   2.317   0.961   0.021   0.756   0.223
B G  1DANH                 27    0.049   3.864  -1.143   0.023   0.655   0.322
B G  1DANH                 28    0.039   3.093  -0.927   0.021   0.756   0.223
B T  1DANH                 29    0.062   8.599  -0.101   0.021   0.756   0.223
B L  1DANH                 30    0.052   9.503   0.559   0.021   0.756   0.223
B I  1DANH                 31    0.063  11.692   0.410   0.021   0.451   0.528
B N  1DANH                 32    0.154  22.546   0.812   0.019   0.141   0.840
E T  1DANH                 33    0.663  92.000   0.748   0.018   0.047   0.935
E I  1DANH                 34    0.344  63.566   1.092   0.018   0.047   0.935
B W  1DANH                 35    0.072  17.340   1.434   0.079   0.592   0.329
B V  1DANH                 36    0.026   4.058   0.955   0.018   0.846   0.136
B V  1DANH                 37    0.026   3.996   1.446   0.011   0.918   0.071
B S  1DANH                 38    0.035   4.149   0.610   0.018   0.846   0.136
B A  1DANH                 39    0.025   2.788   0.557   0.085   0.775   0.140
B A  1DANH                 40    0.037   4.044  -0.692   0.079   0.592   0.329
B H  1DANH                 41    0.087  15.916   0.891   0.079   0.592   0.329
B C  1DANH                 42    0.019   2.710   1.149   0.159   0.516   0.325
B F  1DANH                 43    0.067  13.487   0.583   0.069   0.386   0.545
E D  1DANH                 44    0.371  53.461   0.564   0.020   0.205   0.775
E K  1DANH                 45    0.451  92.812   0.287   0.018   0.088   0.893
E I  1DANH                 46    0.397  73.427  -0.615   0.018   0.047   0.935
E K  1DANH                 47    0.607 124.860  -0.653   0.018   0.047   0.935
E N  1DANH                 48    0.504  73.830   0.220   0.018   0.047   0.935
E W  1DANH                 49    0.272  65.344  -1.269   0.004   0.085   0.910
E R  1DANH                 50    0.478 109.508  -0.621   0.004   0.197   0.799
E N  1DANH                 51    0.347  50.757   1.614   0.004   0.616   0.381
B L  1DANH                 52    0.046   8.459   0.910   0.001   0.900   0.099
B I  1DANH                 53    0.199  36.759   1.776   0.001   0.959   0.040
B A  1DANH                 54    0.021   2.281   1.191   0.001   0.959   0.040
B V  1DANH                 55    0.097  14.878   1.513   0.001   0.959   0.040
B L  1DANH                 56    0.036   6.573  -0.055   0.001   0.959   0.040
B G  1DANH                 57    0.063   4.950  -0.728   0.002   0.816   0.182
E E  1DANH                 58    0.245  42.819   0.145   0.003   0.718   0.279
B H  1DANH                 59    0.181  32.833   0.568   0.003   0.718   0.279
E D  1DANH                 60    0.330  47.524   0.005   0.023   0.655   0.322
B L  1DANH                 61    0.187  34.295  -0.151   0.022   0.552   0.426
E S  1DANH                 62    0.476  55.740  -0.709   0.021   0.451   0.528
E E  1DANH                 63    0.469  81.934   0.239   0.005   0.262   0.733
E H  1DANH                 64    0.549  99.899  -1.089   0.004   0.138   0.858
E D  1DANH                 65    0.663  95.538  -0.766   0.005   0.045   0.951
E G  1DANH                 66    0.723  56.900  -0.095   0.005   0.045   0.951
E D  1DANH                 67    0.474  68.332  -0.643   0.004   0.197   0.799
E E  1DANH                 68    0.413  72.238  -0.451   0.004   0.616   0.381
E Q  1DANH                 69    0.310  55.437   0.777   0.001   0.900   0.099
E S  1DANH                 70    0.476  55.729   0.926   0.001   0.959   0.040
B R  1DANH                 71    0.111  25.488   1.586   0.001   0.959   0.040
E R  1DANH                 72    0.427  97.714   1.398   0.001   0.900   0.099
B V  1DANH                 73    0.029   4.396   0.211   0.001   0.900   0.099
E A  1DANH                 74    0.455  50.196   1.311   0.001   0.900   0.099
E Q  1DANH                 75    0.331  59.081   1.199   0.001   0.959   0.040
B V  1DANH                 76    0.072  11.082   0.631   0.001   0.959   0.040
B I  1DANH                 77    0.125  23.144   0.809   0.001   0.900   0.099
B I  1DANH                 78    0.250  46.268   0.483   0.003   0.718   0.279
B P  1DANH                 79    0.078  10.997   0.914   0.004   0.138   0.858
E S  1DANH                 80    0.560  65.655   0.135   0.005   0.015   0.979
E T  1DANH                 81    0.536  74.343   0.010   0.005   0.015   0.979
B Y  1DANH                 82    0.105  22.438  -0.293   0.005   0.045   0.951
E V  1DANH                 83    0.385  59.144   0.529   0.018   0.047   0.935
E P  1DANH                 84    0.550  77.988  -0.514   0.018   0.047   0.935
E G  1DANH                 85    0.651  51.265  -1.288   0.018   0.019   0.964
E T  1DANH                 86    0.477  66.174  -0.332   0.018   0.019   0.964
B T  1DANH                 87    0.228  31.665   0.371   0.018   0.047   0.935
E N  1DANH                 88    0.481  70.404  -0.400   0.018   0.047   0.935
B H  1DANH                 89    0.106  19.281   0.393   0.018   0.088   0.893
B D  1DANH                 90    0.045   6.470  -0.192   0.022   0.359   0.619
B I  1DANH                 91    0.027   5.088   1.034   0.018   0.846   0.136
B A  1DANH                 92    0.020   2.226   1.137   0.011   0.918   0.071
B L  1DANH                 93    0.022   4.047   1.299   0.011   0.918   0.071
B L  1DANH                 94    0.031   5.713   0.846   0.011   0.918   0.071
B R  1DANH                 95    0.222  50.952   1.701   0.001   0.900   0.099
B L  1DANH                 96    0.038   6.976  -0.164   0.003   0.718   0.279
E H  1DANH                 97    0.517  93.970   1.455   0.021   0.279   0.699
E Q  1DANH                 98    0.537  95.944   1.026   0.020   0.205   0.775
E P  1DANH                 99    0.437  62.010   0.421   0.021   0.279   0.699
B V  1DANH                100    0.068  10.390   0.099   0.021   0.279   0.699
E V  1DANH                101    0.600  92.251   1.440   0.021   0.279   0.699
E L  1DANH                102    0.286  52.348   0.199   0.020   0.205   0.775
E T  1DANH                103    0.353  48.906  -0.491   0.018   0.047   0.935
E D  1DANH                104    0.678  97.700  -0.005   0.018   0.047   0.935
E H  1DANH                105    0.320  58.135   0.647   0.005   0.336   0.660
B V  1DANH                106    0.052   8.008   0.725   0.003   0.718   0.279
B V  1DANH                107    0.243  37.349   1.189   0.002   0.816   0.182
B P  1DANH                108    0.093  13.126   0.226   0.018   0.846   0.136
B L  1DANH                109    0.023   4.120   1.114   0.018   0.846   0.136
B C  1DANH                110    0.066   9.252  -0.548   0.003   0.718   0.279
B L  1DANH                111    0.037   6.683   0.660   0.005   0.336   0.660
B P  1DANH                112    0.069   9.734   0.020   0.005   0.015   0.979
E E  1DANH                113    0.410  71.575   1.019   0.018   0.019   0.964
E R  1DANH                114    0.615 140.766  -0.514   0.018   0.019   0.964
E T  1DANH                115    0.570  79.101  -2.162   0.058   0.017   0.925
B F  1DANH                116    0.288  57.782  -0.866   0.058   0.017   0.925
E S  1DANH                117    0.598  70.062  -0.655   0.053   0.043   0.903
E E  1DANH                118    0.494  86.319  -0.655   0.053   0.043   0.903
E R  1DANH                119    0.591 135.385  -0.781   0.052   0.084   0.864
E T  1DANH                120    0.489  67.783  -0.296   0.052   0.084   0.864
B L  1DANH                121    0.082  15.033  -0.248   0.018   0.047   0.935
E A  1DANH                122    0.448  49.337  -0.218   0.018   0.019   0.964
E F  1DANH                123    0.545 109.462  -0.854   0.018   0.019   0.964
E V  1DANH                124    0.492  75.651  -1.175   0.005   0.015   0.979
B R  1DANH                125    0.208  47.655   0.009   0.004   0.085   0.910
E F  1DANH                126    0.301  60.371   1.615   0.004   0.616   0.381
B S  1DANH                127    0.016   1.910   1.380   0.001   0.900   0.099
B L  1DANH                128    0.089  16.351   1.263   0.001   0.959   0.040
B V  1DANH                129    0.018   2.782   0.949   0.001   0.959   0.040
B S  1DANH                130    0.048   5.579   0.500   0.001   0.900   0.099
B G  1DANH                131    0.037   2.920  -1.751   0.002   0.816   0.182
B W  1DANH                132    0.113  27.225  -0.025   0.022   0.552   0.426
B G  1DANH                133    0.058   4.533  -1.522   0.021   0.451   0.528
E Q  1DANH                134    0.317  56.545   0.605   0.021   0.451   0.528
B L  1DANH                135    0.181  33.178   0.236   0.021   0.279   0.699
E L  1DANH                136    0.454  83.201   0.511   0.019   0.141   0.840
E D  1DANH                137    0.660  95.135  -0.523   0.018   0.047   0.935
E R  1DANH                138    0.707 161.834  -1.419   0.005   0.015   0.979
E G  1DANH                139    0.391  30.795  -1.445   0.005   0.015   0.979
E A  1DANH                140    0.645  71.024  -0.529   0.018   0.019   0.964
E T  1DANH                141    0.354  49.030  -0.224   0.058   0.017   0.925
B A  1DANH                142    0.147  16.144  -0.537   0.181   0.016   0.803
E L  1DANH                143    0.543  99.478   0.153   0.268   0.043   0.689
E E  1DANH                144    0.278  48.497   1.693   0.307   0.165   0.527
B L  1DANH                145    0.067  12.268   0.294   0.231   0.330   0.439
E M  1DANH                146    0.258  51.606   0.987   0.172   0.690   0.138
E V  1DANH                147    0.406  62.448   1.091   0.072   0.855   0.073
B L  1DANH                148    0.034   6.317   0.144   0.011   0.918   0.071
E N  1DANH                149    0.525  76.919   0.561   0.018   0.846   0.136
B V  1DANH                150    0.052   7.977   1.234   0.023   0.655   0.322
E P  1DANH                151    0.416  58.988   0.767   0.023   0.655   0.322
B R  1DANH                152    0.188  43.121   0.169   0.004   0.616   0.381
B L  1DANH                153    0.173  31.731   1.075   0.004   0.616   0.381
E M  1DANH                154    0.460  92.106   1.210   0.004   0.197   0.799
E T  1DANH                155    0.269  37.255   0.821   0.782   0.003   0.216
E Q  1DANH                156    0.752 134.325   1.364   0.923   0.002   0.076
E D  1DANH                157    0.416  59.902   2.309   0.923   0.002   0.076
B C  1DANH                158    0.013   1.867   1.211   0.879   0.010   0.111
E L  1DANH                159    0.363  66.520   1.172   0.879   0.010   0.111
E Q  1DANH                160    0.637 113.679   1.230   0.782   0.003   0.216
B Q  1DANH                161    0.217  38.703   0.673   0.782   0.003   0.216
B S  1DANH                162    0.109  12.775  -0.163   0.782   0.003   0.216
E R  1DANH                163    0.509 116.561  -0.233   0.717   0.014   0.269
E K  1DANH                164    0.572 117.763  -0.520   0.622   0.015   0.363
E V  1DANH                165    0.398  61.203  -1.270   0.339   0.016   0.645
E G  1DANH                166    0.464  36.525  -2.347   0.115   0.016   0.868
E D  1DANH                167    0.519  74.730  -1.356   0.058   0.017   0.925
E S  1DANH                168    0.597  70.004  -1.346   0.018   0.019   0.964
E P  1DANH                169    0.381  54.050  -1.320   0.018   0.019   0.964
E N  1DANH                170    0.430  63.011   1.145   0.018   0.088   0.893
B I  1DANH                171    0.034   6.309  -0.407   0.020   0.205   0.775
E T  1DANH                172    0.250  34.703   1.053   0.018   0.088   0.893
E E  1DANH                173    0.585 102.112  -0.005   0.018   0.019   0.964
B Y  1DANH                174    0.253  54.109  -0.100   0.018   0.047   0.935
B M  1DANH                175    0.030   5.963   0.362   0.021   0.279   0.699
B F  1DANH                176    0.036   7.185   0.339   0.023   0.655   0.322
B C  1DANH                177    0.017   2.359   0.939   0.023   0.655   0.322
B A  1DANH                178    0.028   3.108  -0.092   0.021   0.451   0.528
B G  1DANH                179    0.108   8.468  -1.017   0.021   0.279   0.699
B Y  1DANH                180    0.228  48.681   0.665   0.019   0.141   0.840
E S  1DANH                181    0.585  68.503   0.155   0.018   0.088   0.893
E D  1DANH                182    0.756 108.954   0.015   0.018   0.047   0.935
E G  1DANH                183    0.506  39.799  -1.382   0.018   0.019   0.964
E S  1DANH                184    0.478  55.986  -0.928   0.018   0.047   0.935
E K  1DANH                185    0.436  89.623   0.733   0.019   0.141   0.840
B D  1DANH                186    0.106  15.332  -2.243   0.020   0.205   0.775
B S  1DANH                187    0.125  14.615   0.246   0.020   0.205   0.775
B C  1DANH                188    0.034   4.774  -0.949   0.021   0.279   0.699
E K  1DANH                189    0.343  70.617   0.314   0.020   0.205   0.775
B G  1DANH                190    0.206  16.181  -1.557   0.019   0.141   0.840
B D  1DANH                191    0.215  31.025  -0.037   0.018   0.047   0.935
B S  1DANH                192    0.241  28.245  -1.874   0.018   0.019   0.964
B G  1DANH                193    0.203  15.960  -2.400   0.018   0.047   0.935
B G  1DANH                194    0.116   9.145  -0.552   0.018   0.088   0.893
B P  1DANH                195    0.107  15.112  -0.846   0.021   0.279   0.699
B H  1DANH                196    0.077  14.043   0.404   0.004   0.616   0.381
B A  1DANH                197    0.080   8.794  -0.142   0.018   0.846   0.136
B T  1DANH                198    0.052   7.212   0.327   0.018   0.846   0.136
E H  1DANH                199    0.442  80.363   0.525   0.004   0.616   0.381
E Y  1DANH                200    0.557 118.967  -1.166   0.004   0.197   0.799
E R  1DANH                201    0.750 171.704  -0.285   0.005   0.015   0.979
E G  1DANH                202    0.548  43.104  -1.289   0.005   0.015   0.979
E T  1DANH                203    0.327  45.410   1.695   0.004   0.514   0.481
B W  1DANH                204    0.109  26.214   0.986   0.001   0.900   0.099
B Y  1DANH                205    0.122  26.007   1.778   0.001   0.959   0.040
B L  1DANH                206    0.029   5.347   0.274   0.001   0.959   0.040
B T  1DANH                207    0.054   7.490   1.191   0.006   0.962   0.032
B G  1DANH                208    0.033   2.605  -0.437   0.011   0.918   0.071
B I  1DANH                209    0.038   7.030   0.645   0.011   0.918   0.071
B V  1DANH                210    0.040   6.102   0.472   0.021   0.756   0.223
B S  1DANH                211    0.046   5.379  -0.391   0.021   0.756   0.223
B W  1DANH                212    0.147  35.257   0.021   0.021   0.451   0.528
B G  1DANH                213    0.111   8.736  -1.019   0.064   0.216   0.721
E Q  1DANH                214    0.389  69.475   0.626   0.113   0.087   0.800
E G  1DANH                215    0.370  29.095  -0.776   0.113   0.087   0.800
B C  1DANH                216    0.070   9.898  -0.439   0.113   0.043   0.844
B A  1DANH                217    0.270  29.710  -1.006   0.058   0.017   0.925
E T  1DANH                218    0.495  68.712   0.827   0.058   0.017   0.925
E V  1DANH                219    0.585  89.961   0.574   0.018   0.019   0.964
E G  1DANH                220    0.693  54.547  -0.732   0.003   0.003   0.994
E H  1DANH                221    0.306  55.734   1.135   0.005   0.015   0.979
B F  1DANH                222    0.149  29.864  -0.632   0.018   0.019   0.964
B G  1DANH                223    0.064   5.005  -1.771   0.022   0.359   0.619
B V  1DANH                224    0.044   6.732   0.088   0.023   0.655   0.322
B Y  1DANH                225    0.107  22.909   0.114   0.085   0.775   0.140
B T  1DANH                226    0.047   6.505   0.441   0.079   0.592   0.329
B R  1DANH                227    0.238  54.479   0.552   0.148   0.418   0.435
B V  1DANH                228    0.046   7.132   0.338   0.502   0.102   0.396
E S  1DANH                229    0.307  35.957   0.261   0.622   0.015   0.363
E Q  1DANH                230    0.360  64.350   0.984   0.717   0.014   0.269
B Y  1DANH                231    0.062  13.271   0.306   0.879   0.010   0.111
B I  1DANH                232    0.210  38.869   1.125   0.938   0.007   0.055
E E  1DANH                233    0.563  98.409   1.420   0.970   0.001   0.030
B W  1DANH                234    0.104  25.060   0.988   0.970   0.001   0.030
B L  1DANH                235    0.021   3.918   0.937   0.988   0.000   0.012
E Q  1DANH                236    0.372  66.368   1.584   0.988   0.000   0.012
E K  1DANH                237    0.525 107.931   1.735   0.970   0.001   0.030
B L  1DANH                238    0.166  30.303   1.529   0.970   0.001   0.030
B M  1DANH                239    0.053  10.685   0.329   0.923   0.002   0.076
E R  1DANH                240    0.573 131.194   0.830   0.782   0.003   0.216
E S  1DANH                241    0.691  81.044   0.099   0.502   0.002   0.495
E E  1DANH                242    0.459  80.187  -0.763   0.109   0.005   0.886
E P  1DANH                243    0.517  73.419  -2.028   0.016   0.005   0.979
E R  1DANH                244    0.500 114.523  -0.517   0.005   0.015   0.979
E P  1DANH                245    0.481  68.311  -1.243   0.005   0.015   0.979
E G  1DANH                246    0.465  36.588  -2.243   0.005   0.045   0.951
B V  1DANH                247    0.235  36.043  -1.233   0.004   0.085   0.910
E L  1DANH                248    0.328  60.038  -0.935   0.004   0.085   0.910
E L  1DANH                249    0.416  76.133  -1.568   0.005   0.045   0.951
E R  1DANH                250    0.542 124.164  -1.497   0.005   0.015   0.979
E A  1DANH                251    0.380  41.865  -1.782   0.003   0.003   0.994
E P  1DANH                252    0.443  62.918  -2.025   0.003   0.003   0.994
E F  1DANH                253    0.500 100.390  -1.019   0.003   0.003   0.994
E P  1DANH                254    0.729 103.459  -1.532   0.000   0.000   1.000

Explain the output.  Go back.
```

---
